# Multiple sclerosis in the Arabian Gulf countries: a consensus statement

**DOI:** 10.1007/s00415-013-6876-4

**Published:** 2013-03-17

**Authors:** Saeed Bohlega, Jihad Inshasi, Abdel Rahman Al Tahan, Abu Bakr Madani, Hussien Qahtani, Peter Rieckmann

**Affiliations:** 1 King Faisal Specialist Hospital and Research Center, Riyadh, Kingdom of Saudi Arabia; 2 Rashid Hospital, Dubai, United Arab Emirates; 3 King Khaled University Hospital, Riyadh, Kingdom of Saudi Arabia; 4 King Khaled National Guard, Jeddah, Kingdom of Saudi Arabia; 5 Academic Hospital Bamberg, University of Erlangen, Erlangen, Germany

**Keywords:** Multiple sclerosis, Diagnosis, Treatment, Guidelines, Arabian Gulf

## Abstract

The epidemiology of multiple sclerosis (MS) is rapidly changing in many parts of the world. Based on the Kurtzke classification, the Arabian Gulf Region is located in a low-risk zone for MS; however, recent studies suggest a moderate-to-high prevalence nearby (31–55 MS per 10,0000 individuals), with an increase in incidence in recent years. The relapsing-remitting disease course ratio is 2.5:1 versus the primary progressive type. In a geographic area that was previously associated with low prevalence; the recent high prevalence and fast rising incidence of MS in the gulf countries, encouraged the neurologists of this region to meet in a consensus panel, in order to share our latest findings in terms of MS epidemiology and consent on MS management in the Arabian Gulf. Therefore 20 key opinion leader neurologists and MS experts representing various countries of the Arabian Gulf have met in Dubai on the 2 and 3 February 2012, they shared their latest epidemiological findings, discussed recent MS aspects in the region, and consented on MS management relevantly to this geographic area.

## Epidemiology of MS in the region

There are relatively few studies regarding epidemiology of MS in this region [1]. In 1988, Yaqub et al. [2] published a paper about MS in Saudi Arabia, stating that there are indications of increasing incidence of MS in Saudi Arabia. They noted that the symptomatology of MS and the site of lesions are similar to that seen in the West, but the course and evolution might be different.

Ten years later, Daif et al. [3] published another paper about the pattern of presentation of multiple sclerosis (MS) in Saudi Arabia, stating also that it resembles the western type of MS.

In an unpublished communication, Prof. Bohlega estimated the prevalence of MS in Saudis to be 40/100,000 in 2008. “Although it used to be thought that MS is not common in Saudi Arabia, it is now clear that it is fairly prevalent, under-diagnosed and in increase”, stated Prof. Bohlega.

In their retrospective study in 2005, Alshubaili et al. [1] examined the changes in incidence and prevalence of MS in Kuwait. The total incidence rate increased from 1.05/100,000 population in 1993 to 2.62/100,000 in 2000. The increased incidence of MS was most pronounced among Kuwaiti women (from 2.26/100,000 in 1993 to 7.79/100,000 in 2000. The total prevalence rate increased from 6.68/100,000 in 1993 to 14.77/100,000 in 2000. It was much higher for Kuwaitis (31.15/100,000), as compared to non-Kuwaitis (5.55/100,000), in a complete reversal of the pattern observed before 1990. The prevalence was also higher among Kuwaiti women (35.54/100,000), as compared with Kuwaiti men (26.65/100,000). In conclusion, the incidence and prevalence of MS in Kuwait has increased between the early and late 1990s with no signs of leveling off.

In a recent paper in 2011, Inshasi and Thakre [4] determined the prevalence of MS in Dubai (UAE). They found the prevalence to be 54.77/100,000 in 2007 which was surprisingly high. There were no previous studies to compare to it. They concluded that Dubai should be considered as one of the regions with medium to high risk of MS, with a prevalence rate higher than what has been previously believed.

So, why is MS prevalence increasing?

Several hypotheses attempt to answer this question but none of them is proven. Is it the increase in the young population? Is it the change in lifestyle of this region, with the introduction of the air-conditioning systems in the region? Is it the vitamin D deficiency?

In fact, vitamin D deficiency has been recently noted in the Gulf region despite the area’s sunny climate. Although our countries have a sunny environment, vitamin D deficiency is one of the main public health problems. Studies in Saudi Arabia revealed that 28 to 80 % of adults had vitamin D deficiency [5].

Is it consanguinity? Knowing that, in the Arabian Gulf countries every other marriage is consanguineous, in 2011 Al Jumah et al. [6, 7] correlated the prevalence of familial multiple sclerosis (FMS) and rate of parental consanguinity (PC). He concluded that MS patients with a history of PC were more likely to have FMS, suggesting a potential role of consanguinity.

With lack of official registries and published studies in some countries concerning the epidemiology of MS in the region, a central MS registry and long term follow-up epidemiological studies are recommended [4].

## MS management in the Arabian Gulf countries

### Diagnosis

Successful management of MS requires early intervention. Knowing that, permanent axonal loss begins before MS is diagnosed, and treatment is more effective in the inflammatory stage, when there are more intact axons to protect. And the famous quote “delaying treatment in MS: what is lost is not regained” is always true [8]. Therefore, early intervention requires early diagnosis, and the 2010 McDonald criteria promote early diagnosis. And we neurologists of this region use the McDonald 2010 criteria for diagnosing MS, adding complementary tests to rule out other likely diagnosis of vasculitis, Behcet disease, brucellosis and B12 deficiency which are more prevalent in our countries than in the western countries.

We agree that MRI is the best imaging technology for detecting the presence of MS plaques or lesions in different parts of the CNS. However, the diagnosis of MS cannot be made solely on the basis of the MRI, the patient’s medical history and the neurologic exam can provide enough evidence to meet the diagnostic criteria. Cerebrospinal fluid analysis is less used because the patients are usually reluctant to undergo a lumbar puncture.

We would like to emphasize that the diagnosis of MS should be made by a neurologist and not by other specialists as it is common in our region. And therefore, after the diagnosis of MS is established, we recommend that the patient should be regularly followed-up by an expert neurologist, more frequently after the early phase of diagnosis.

### Treatment

Several therapies for MS exist, although there is no known cure. The most common initial course of the disease is the relapsing-remitting subtype.

As with any medical treatment, medications used in the management of MS may have several adverse effects, and many possible therapies are still under investigation. At the same time, different alternative treatments are pursued by many patients, despite the paucity of supporting, comparable, replicated scientific study. We state here the Zamboni liberation procedure is not an option for MS treatment.

### Disease-modifying treatments

As of 2012, six disease-modifying treatments have been approved by regulatory agencies of different countries, including the U.S. Food and Drug Administration (FDA) and the European Medicines Agency (EMA). The six drugs are interferon beta-1a (Avonex, Rebif), interferon beta-1b (Betaferon, Extavia), glatiramer acetate (Copaxone), mitoxantrone (Novantrone), natalizumab (Tysabri) and fingolimod (Gilenya), the first oral drug available. In our region all interferons are available for the treatment of MS.

The aim of starting disease-modifying treatment is to control relapses, to slow the accumulation of the disease on MRI, the disability progression, and finally to improve the quality of life of the patient.

#### Clinically isolated syndrome (CIS)

The earliest clinical presentation of relapsing-remitting MS (RRMS) is the clinically isolated syndrome (CIS), i.e., a single attack of a single symptom. During a CIS, there is a subacute attack suggestive of demyelination but the patient does not fulfill the criteria for diagnosis of MS [9]. Several studies have shown that early treatment of CIS after the initial presentation can delay the development of clinically definite multiple sclerosis (CDMS). These results support the use of interferon after a first clinical demyelinating event and indicate that there may be beneficial effects of immediate treatment compared with delayed initiation of treatment [10–12]. Therefore, we agree that patients who present with CIS should be treated. However, CIS patients who do not wish to start treatment should be followed up by MRI at 3 month intervals. It should be stated that many cases of CIS are reclassified as definite MS, according to final amendment of McDonald Criteria 2010, and should be treated accordingly. Therefore, all neurologists at this meeting support early initiation of IFNβ therapy in patients with CIS in view of the supportive data and its availability in the region. However, in CIS patients with normal or few lesions on brain MRI and especially those with monofocal symptoms and complete recovery, a brief watchful phase with a follow up brain MRI at 3–6 months is appropriate. Also recently glatiramer (trade name Copaxone) has been shown to be beneficial after a first clinical demyelinating event; however Copaxone is unavailable in our region [13].

#### Relapsing-remitting MS (RRMS)

The two approved interferons are the interferon beta-1a (with two commercial formulations, with trade names *Avonex* and *Rebif*; the first injected weekly, the latter three times a week), and the interferon beta-1b (trade name *Betaferon, Extavia*), injected every other day.

The other approved drugs are glatiramer acetate or Copaxone, injected daily, which is a mixture of polypeptides which may protect important myelin basic proteins by substituting itself as the target of immune system attack [14]. We note here, that Copaxone is not available in our region. Mitoxantrone is an immunosuppressant also used in cancer chemotherapy. Natalizumab, marketed as *Tysabri* is a monoclonal antibody and finally fingolimod (trade name Gilenya) is a sphingosine-1-phosphate receptor modulator.

Mitoxantrone use is limited by severe cardiotoxicity, and it is not considered as a long-term therapy. Recent data show higher risk of leukemia, almost to exclude its use in RRMS in the presence of fingolimod and natalizumab [15, 16].

Worth noting, that neither fingolimod nor natalizumab have had a head to head comparison with high dose beta interferon.

The population of beta interferon studies is different from that of fingolimod and natalizumab (higher EDSS and late MS/interferon group vs. lower EDSS and early MS/other group).

All six approved medications differ in their efficacy rate and for some studies of their long-term effects are still lacking.

The longest assessment of any MS-specific treatment is the 21-year long-term follow-up study with *Betaferon* (interferon beta-1b). It provides the first strong survival evidence for MS treatment, and further supports the importance of starting patients as soon as possible on an effective disease-modifying therapy with a favourable safety profile in the long-term.

There is a strong body of clinical trial evidence that dose and frequency of administration are important to achieve optimal clinical benefit in MS. These data suggest that higher dose and more frequent dosing of interferon beta result in greater efficacy. This finding was confirmed by the results of INCOMIN and EVIDENCE. Results from a pilot study in patients with RRMS have indicated that increasing the dose of IFNbeta-1b to 500 µg (16 MIU) had a more pronounced biological effect compared with the standard 250 µg dose [17].

Therefore, we consider that there is overwhelming evidence that high dose/high frequency betaferon is recommended in RRMS, but the final decision is based on agreement between the informed and educated patient and the neurologist, in order to ensure long term patient compliance and adherence to the treatment.

#### Primary progressive MS

At this time, there is no FDA-approved treatment for PPMS. Research studies usually focus on medications for the relapsing forms of MS. There have only been a handful of treatment studies specifically for PPMS; the results so far have not shown a significant treatment effect. The standard FDA-approved medications for MS (interferons, glatiramer acetate, mitoxantrone, natalizumab) have not been proven useful in slowing the progression of PPMS.

We encourage people with PPMS to maintaining mobility and fitness. In addition, there are medications which may be used to treat symptoms such as bladder and bowel urgency, erectile problems, spasticity, and pain, if such treatments are needed.

Occasionally, intermittent (on and off) intravenous (IV) steroids have been tried in patients with primary progressive MS. Such therapies have provided only limited results in these cases. Also, the chemotherapeutic drug methotrexate has been given in weekly oral doses to patients with PPMS.

#### Assessing response to therapy

The group generally agreed that treatment response should be evaluated at 6–12 months intervals depending on accessibility of MRI which differs from one country to another. Poor response is defined by the presence of at least two of the following:

1. One or more disabling relapses in the previous year
2. An active MRI as defined by the presence of two or more new T2 W/Gd + lesions
3. Sustained increase in EDSS by one step (for EDSS ≤ 5.5) or half a step for EDSS ≥ 6.0.

In case of treatment non-response in RRMS, patients are advised to switch from first line to second line agents. The group generally classifies IFNβ as first-line agents in view of their well-established benefit/risk profiles over both the short and the long-term. Most neurologists in our region follow the EMA indication which recommends the use of natalizumab and fingolimod as second-line agents in case of treatment failure with IFNβ [18]. Fingolimod was approved as a first-line agent in the US, but there is still some reluctance to use it as such in the Arabian Gulf region due to potential concerns about its safety profile and lack of long term safety follow-up. As mentioned previously, and based on clinical evidence from the INCOMIN and EVIDENCE trials suggesting that higher dose and more frequent dosing of IFNβ results in greater efficacy, the group considers moving a non-responder patient on low dose interferon to high dose IFNβ as an appropriate option before escalation to second- line agents.

#### Duration of treatment

Knowing that there is no known cure for MS at this time, beta interferon therapy should be maintained on a long term basis in order to maintain the stability of the disease. But if the patient is not responding to the treatment, we will consider escalating therapy with the second line treatments.

Escalation therapy is considered when the patient satisfies criteria for non-responders [19]. In this case, we consider escalation therapy with the new therapies, the oral fingolimod or the intravenous natalizumab.

#### Vitamin D supplement

More than 30 years have passed since vitamin D was originally hypothesized to be an important environmental determinant of the prevalence of MS. During the three decades following the initial linking of vitamin D and MS, evidence has continued to mount. It is now known that MS occurs more frequently in individuals with lower blood levels of vitamin D. A study found that, compared to those with the highest vitamin D blood levels, those with the lowest blood levels were 62 % more likely to develop MS. A recent study has quantified the impact of vitamin D blood levels on risk for MS relapse—for each 4 ng/mL increase in 25-hydroxy vitamin D in the blood; the risk for MS relapse is reduced by 12 %. In a randomized controlled trial, supplementation with doses of vitamin D ranging from 10,000 to 40,000 IU daily over the course of 52 weeks resulted in a reduction in relapses and a reduction in the number of aggressive immune cells in patients with MS [20].

We should not be surprised if vitamin D emerges as a frontline treatment for MS in the coming years. However, instead of waiting for mainstream physicians to begin recommending vitamin D to MS patients, and being aware of the high prevalence of vitamin D deficiency in our region, we suggest that all MS patients monitor their blood levels of 25-hydroxyvitamin D and maintain a blood level of 50–80 ng/mL. The amount of supplementation required to achieve this blood level varies from one person to another, but it appears that many individuals require supplementation of 5,000–8,000 IU of vitamin D each day to reach these levels. Supplement with vitamin D may reduce the risk of conversion from a first clinical event suggestive of MS to clinical definite MS, as well as reduce the relapse rate among patients with relapsing remitting MS.

## Conclusion

Latest evidence from epidemiological studies have indicated that the Arabian Gulf region has a high prevalence of MS. Based on these facts we aim for a better MS awareness in our region, and we look forward to patient education that should be made by the neurologist and follow up by a trained MS nurse in order to enhance patient’s adherence to treatment and his/her quality of life.
